# Editorial: Present and future of biological fluid biomarkers in dementia

**DOI:** 10.3389/fnagi.2023.1331799

**Published:** 2023-11-20

**Authors:** Javier Frontiñán-Rubio, Yoana Rabanal-Ruiz, Juan R. Peinado, Tomas Deierborg

**Affiliations:** ^1^Oxidative Stress and Neurodegeneration Group, Medical Sciences Department, Medical School, UCLM, Ciudad Real, Spain; ^2^Regional Centre for Biomedical Research, University of Castilla-La Mancha, Ciudad Real, Spain; ^3^Instituto de Investigación Sanitaria de Castilla-La Mancha (IDISCAM), Toledo, Spain; ^4^Department of Experimental Medical Science, Experimental Neuroinflammation Laboratory, BMC, Lund University, Lund, Sweden

**Keywords:** Alzheimer's disease, biomarkers, saliva, plasma, CSF, urine

Alzheimer's disease (AD) and dementia constitute significant challenges to global healthcare systems, as a result of increasing life expectancy that leads to a larger population of the elderly. To implement timely interventions and improve the patient outcome, it is crucial the establishment of approaches for an early and accurate diagnosis of these neurodegenerative disorders. In this context, recent advances have shed light on the potential usefulness of fluid biomarkers (Simrén et al., 2023) and their potential impact has been supported by the significant increase of publications that address this field. This topic explores the advantages, limitations, and prospects of fluid biomarkers, including those found in cerebrospinal fluid (CSF), blood, urine, and saliva.

Development of fluid biomarkers started with the use of CSF due to its proximity to the brain parenchyma and CSF biomarkers have been shown to be generally helpful in detecting AD's preclinical and symptomatic stages (McGrowder et al., 2021; Simrén et al., 2023). To date, approximately 8,000 studies have been published evaluating these biomarkers, one-third of which were published as of 2020 (Figure 1), to specifically detect beta-amyloid (Aβ) and phosphorylated tau protein levels. The levels of 42 amino acids long Aβ peptides (Aβ42) were used as a biomarker of potential interest; however, the measurement of the Aβ42/40 ratio has been recently considered to be a more accurate parameter which inversely correlates with the extent of the Aβ plaque deposition (Simrén et al., 2023). Additionally, the levels of phosphorylated threonine species 181 (p-tau181) and 231 (p-tau231), despite offering a high hit rate, can be altered in older adults without AD symptoms. Therefore, it is essential to consider the potential use of other CSF protein markers to provide a more accurate diagnosis (Pedrero-Prieto et al., 2020, 2021; McGrowder et al., 2021). In this regard, in the present Research Topic, Lu highlights the study of 14-3-3-3ζ protein levels as a potential use in AD diagnosis and prognosis.

**Figure 1:**
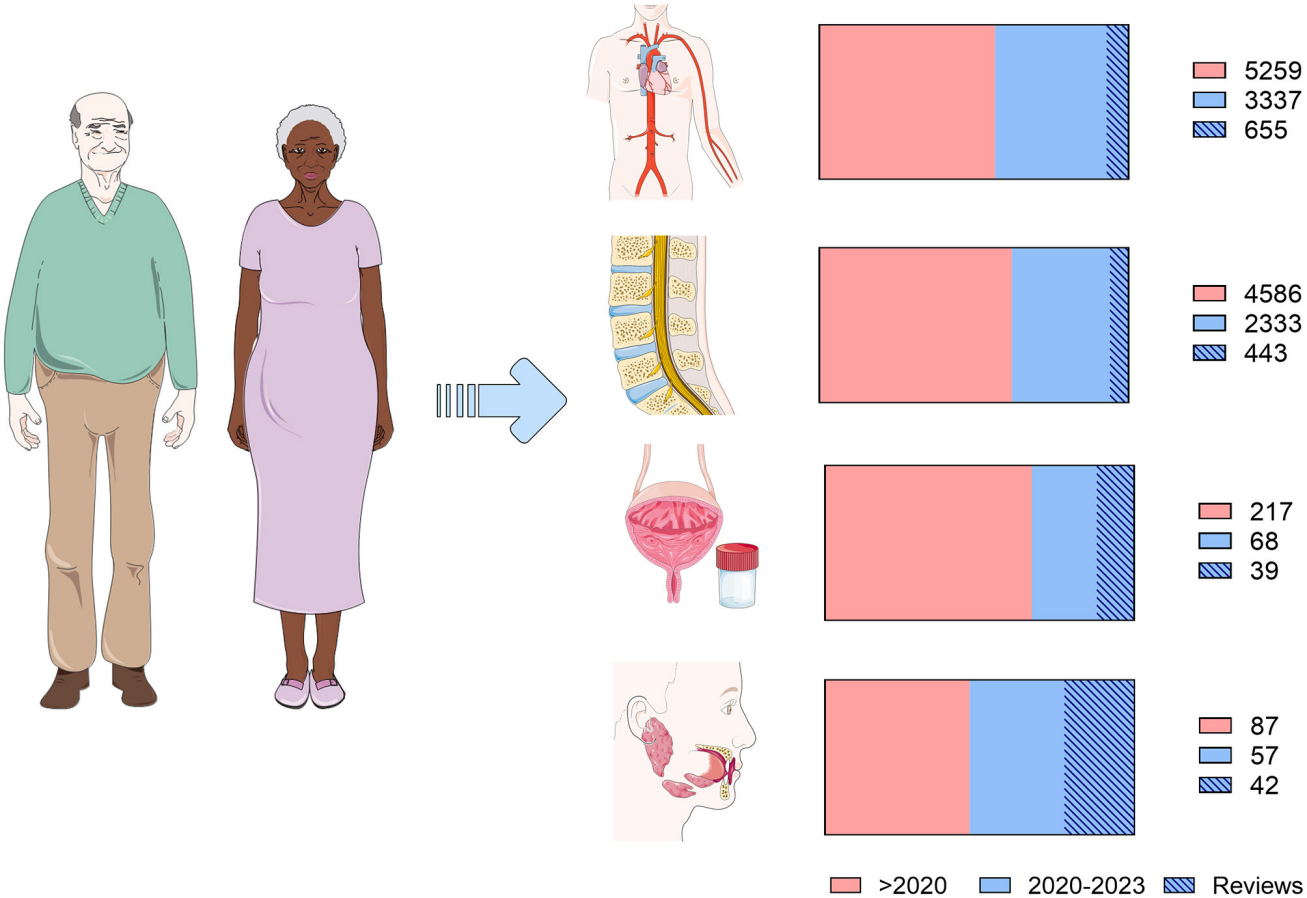
Present of biological fluid biomarkers in Alzheimer's disease and dementia. Diagram representing published articles on fluid biomarkers. A search was performed in the PubMed database according to the following search patterns for the four biofluids analyzed in this article: (Alzheimer's disease OR Dementia) AND biomarkers AND (blood OR plasma OR serum); (Alzheimer's disease OR Dementia) AND biomarkers AND (cerebrospinal fluid OR CSF); (Alzheimer's disease OR Dementia) AND biomarkers AND Saliva; (Alzheimer's disease OR Dementia) AND biomarkers AND urine. Articles have been categorized into those published before (pink) and after 2020 (blue). Within these, those corresponding to reviews and systematic reviews are marked. Articles published up to August 25, 2023, have been included.

Notwithstanding this, CSF sample collection requires invasive procedures, which reinforces the need to explore biomarkers from more accessible biofluids, which would allow studies to be carried out with more subjects. In this sense, blood-based biomarkers exhibit great potential. Despite the fact that more than 7,000 articles have been published on this topic (Figure 1), the potential biomarkers show very low blood concentrations, which makes reliable detection difficult. This explains why the search for biomarkers in blood has currently failed, although the emergence of new and more sensitive techniques has opened the door to promising new research in this field (Simrén et al., 2023). Indeed, studies with large cohorts have shown a good correlation between plasma and CSF in biomarkers such as pTau, Aβ, or the neurofilament light chain (Nfl) (Teunissen et al., 2022). A recent study has reported the potential role of brain-derived plasma tau in the specific diagnosis of AD (Gonzalez-Ortiz et al., 2023). And, in this Research Topic, Chen L. et al. report a systematic review illustrating how p-tau217 is a promising plasma biomarker to differentiate mild cognitive impairment (MCI) and AD. Also, differentiation of neuroinflammatory components, e.g., TREM2 and galectin-3, can be important to understand both the pathogenesis *per se* as well as responsiveness in clinical trials (Boza-Serrano et al., 2019, 2022).

Additionally, in recent years, increased efforts have been made to identify biomarkers in other easily accessible fluids, which would allow frequent monitoring and repeated measurement without affecting the patient's quality of life. Among them, urine is particularly relevant and several promising studies in this fluid have been published the past few years (Seol et al., 2020). They are addressed to identify metabolically derived compounds that differentially appear in the urine of patients with distinct types of dementia. Formaldehyde appears to be one of the most promising compounds and the meta-analysis published in this Research Topic carried out by Chen F. et al., describes how the levels of this metabolite are increased in the urine of AD patients. However, given the complexity of urine composition and the lack of accurate detection techniques, further studies are still needed to implement these biomarkers in daily clinical practice.

Saliva is framed in a similar scenario to that of urine since this biofluid exhibits great diagnostic potential. However, it still requires technical development for the identification of specific valuable biomarkers. This straightforward, inexpensive, non-invasive biofluid contains proteins and metabolites related to the development of different types of dementia. In the Research Topic “*Present and future of biological fluid biomarkers in dementia*” two promising papers have been published on the potential use of saliva as a source of biomarkers. McNicholas et al. present a panel of inflammatory proteins whose levels are differentially expressed in saliva of MCI, AD, and cognitively normal patients. This pilot study carried out in Australia yields auspicious results. Additionally, the results presented by Marksteiner et al. also highlight saliva's role for AD diagnosis and prevention. Using Lumipulse technology, in this pilot trial, the authors identify detectable total tau and ptau181 levels in the saliva of patients with AD and MCI and observe a differential representation between both pathological conditions. McNicholas et al. and Marksteiner et al. studies offer promising results that need to be replicated in large cohorts, but they could establish the first steps toward using a biofluid with ideal conditions to diagnose and monitor neurodegenerative pathologies.

While CSF currently constitutes the most reliable and widely used biofluid to diagnose dementia since it provides a direct window into the brain, efforts are underway worldwide to identify reliable biomarkers in other fluids with easier access and handling. In this regard, plasma constitutes the option that offers the most reliable results. However, new biofluids are emerging as novel candidates. Based on the results presented in this Research Topic, saliva may be ideal to solve one of the most significant problems of AD and other dementias, its early diagnosis. Undoubtedly, we are living in a fascinating time wherein new biomarkers, techniques and fluids offer more hopeful results daily.

## Author contributions

JF-R: Conceptualization, Investigation, Methodology, Visualization, Writing – original draft, Writing – review & editing. YR-R: Conceptualization, Investigation, Writing – original draft, Writing – review & editing. JP: Supervision, Validation, Writing – review & editing. TD: Supervision, Validation, Writing – review & editing.
